# Evaluation of the Psychiatric Disorders among Amphetamine Addicts in Rehabilitation Centers: A Cross-Sectional Analysis

**DOI:** 10.1155/2024/1643693

**Published:** 2024-08-06

**Authors:** Saud D. AlOtaibi, Hossam A. Elsisi, Mohammed J. AlShammary, Saud A. AlQader, Hejab A. AlHarbi, Bayan R. AlOlaiyan, Ahmad O. Alanazi, Firas S. AlMendeel, Yazeed N. AlHarbi, Ibrahim AlKhalaf, Ahmad H. Alhowail, Abdelhamid Mohamed Elwy, Ashraf M. Emara

**Affiliations:** ^1^ Department of Pharmacology and Toxicology College of Pharmacy Qassim University, Buraydah 51452, Qassim, Saudi Arabia; ^2^ Pharmaceutical Care Services King Saud Medical City Ministry of Health, Riyadh 12746, Saudi Arabia; ^3^ Qassim Health Cluster Ministry of Health, Qassim, Saudi Arabia; ^4^ Department of Clinical Pharmacology Faculty of Medicine Zagazig University, Zagazig 44519, Egypt; ^5^ Mental Health Hospital ERADAH, Sakaka 72385, Jouf, Saudi Arabia; ^6^ Mental Health Hospital ERADAH, Buraydah, Qassim, Saudi Arabia; ^7^ Qassim National Hospital, Buraydah, Qassim, Saudi Arabia; ^8^ Department of Forensic Medicine and Clinical Toxicology Faculty of Medicine Tanta University, Tanta, Egypt

## Abstract

**Background:**

People who are addicted to amphetamines have a much greater chance of developing psychosis compared to those who are not. It is essential to study the behavioral and psychological effects of amphetamines. Therefore, this research aimed to examine conditions such as depression, anxiety, mood, cognitive abilities at the workplace, and social responsibilities by using sociodemographic factors as useful tools in determining effective strategies for preventing, managing, and treating amphetamine addiction.

**Methods:**

A cross-sectional study among addicts hospitalized at two rehabilitation centers across Saudi Arabia between May and October 2023. A validated questionnaire consisting of psychiatric disorders assessment tools was distributed to healthcare professionals to start an interview with addicts to assess the abnormalities. The results were compared with healthy people (control). The assessment tools used are Hamilton Anxiety and Depression Rating Scale, Young Mania Rating Scale, and Work and Social Adjustment Scale. The data were analyzed using SPSS version 22.0. Pearson correlation coefficients (*r*) were employed.

**Results:**

A total of 60 subjects participated in this study. The participants were divided into two groups (*n* = 60): group I was control (*n* = 25) healthy volunteers and group II was amphetamine abusers (*n* = 35), who were hospitalized for detoxification. The ages ranged from 18 to 60 years old with mean ages of 38.68 (±8.14) and 37.77 (±10.95) years in the control and amphetamine groups, respectively. Among the addicts, the mean severity dependence scale value was 10.46 (±1.82), which denotes high dependency on the illicit drug. The prevalence of high levels of anxiety, depression, and bipolar disorder was significantly higher among addicts when they were compared to healthy people (control). The assessment of the Work and Social Adjustment Scale (WSAS) reflected a higher impairment that minimized their ability to perform the work requirements, home management, social leisure, and relationships.

**Conclusions:**

The addiction to amphetamines was associated with high impairment of work performance and social obligations and a negative impact on the addict's mental health. The risk of suffering anxiety, depression, and bipolar is higher than in nonaddict people. These effects are attributed to brain damage, neurotoxicity, and neuronal inflammation, particularly when these substances are abused over extended periods and at higher doses.

## 1. Introduction

Amphetamines are the second most widely abused class of illicit drugs after cannabis in the world and are considered as extremely addictive drugs. These drugs act on the mesolimbic dopaminergic neurons of the reward system by inducing the release of dopamine. Also, these drugs induce the release of norepinephrine in the nucleus accumbens in the synaptic clefts. Amphetamines may induce the release of serotonin and can affect the reuptake [1, 2]. Psychosis triggered by amphetamine in around 30% of abusers may lead to a permanent disorder for the length of life. Derivatives of amphetamines are still in use in the clinics to treat narcolepsy and hyperactivity due to attention deficit disorder. Amphetamines can inhibit the reuptake of monoamines such as dopamine, epinephrine, norepinephrine, and serotonin, which leads to a rise in the level within the neuronal synapse. Amphetamines else can block the vesicular monoamine transporters which can result in the enhanced nonvascular release of monoamines. The release of norepinephrine and dopamine in the nucleus accumbens increases the feeling of euphoria and induces a reward feedback loop which may cause an addiction. Psychiatric symptoms induced by amphetamines are a result of direct mechanisms such as sensitization of the dopaminergic system, high level of dopamine inside the midbrain and forebrain, the toxicity of glutamate and GABA system, damage inside the cortical and subcortical area, and the dysregulation of neuronal networks inside the brain [3–5]. The involvement of indirect mechanisms to induce psychosis is proven in the literature such as long-term abuse, high doses, genetic factors, sleep deprivation, and reactive oxygen species (ROS). Other mechanisms implicated in amphetamine-induced psychosis include glutamate alterations, neuronal inflammation and apoptosis, and neurotoxicity. The function of dopamine and its sensitization are confirmed in the process of developing schizophrenia and being involved in psychotic status which is triggered by abused drugs [4]. According to a meta-analysis which includes 59 studies, the study investigated the psychiatric disorders among addicts, and amphetamine abuse was associated with higher odds of psychosis, and there is a high risk of experiencing violence, depression, and even suicidality [6]. Another study confirmed that there was a significant association between methamphetamine abuse and depression. The addicts who were diagnosed with a methamphetamine use disorder had a higher incidence of depression in comparison with those who did not abuse this substance [7].

Bipolar disorder is a mental illness that causes dramatic, uncontrollable mood swings, and patients may feel bouts of mania and depression, which can affect sleep habits, energy, and impair behavior and judgment [8]. Stimulants can cause suicidal ideation during episodes of bipolar disorder's depressive or mania and trigger mood swings, which may promote the intensity of symptoms. Bipolar disorder patients who abuse drugs may be more likely to harm themselves. Since stimulants such as amphetamines can cause mania even in people without bipolar disorder, amphetamine abuse can severely worsen the status of mental health in people who have the bipolar spectrum. Stimulant drugs at high doses can induce the symptoms of mania and trigger a psychosis that is highly similar to those of bipolar or schizophrenic illnesses. These symptoms generally resolve within 2 days after discontinuation of the stimulant, although some reports claim that symptoms may last for 6 days or longer as has been reported by research [9–11].

Compared to schizophrenic psychosis, amphetamine-induced acute psychosis appears to demonstrate a rapid recovery. It also seems to resolve with substance abstinence; however, this recovery may be sometimes incomplete as have been seen by experts [12]. Japanese research suggests that amphetamine-induced psychosis may require years to recover from. Another report claimed that amphetamine abuse has been accompanied by complications such as anxiety, loss of memory, depression, psychosis, sleep deprivation, neurological deficits, another coingestion substance, and social dysfunction [11–13].

There are limited studies that assessed and detailed the psychiatric disorders among amphetamine abusers, the intensity, and complications in the work and social demands. Therefore, it is a fundamental reason to start this research to assess how extent of the changes in the personality, attitude, behavior, and cognitive status following the amphetamine abuse.

### 1.1. Aim of Study

This study aims to investigate the presence and severity of psychiatric disorders such as depression, mood, and anxiety disorders in amphetamine abusers, hospitalized in rehabilitation centers (Al-Amal Hospital for Mental Health, KSA), to evaluate and grade the severity of the psychiatric disorders among the participants and to investigate the distribution of the problem across various sociodemographic divides.

## 2. Methods

### 2.1. Study Design

A cross-sectional study was conducted among addicts at government rehabilitation centers of healthcare facilities across Saudi Arabia between May and October 2023. A validated questionnaire consisting of psychiatric assessment tools was distributed to healthcare professionals to start an interview with addicts to assess the illness. The assessment tools used are the Hamilton Anxiety Rating Scale (HAM-A), Hamilton Depression Rating Scale (HAM-D), Young Mania Rating Scale (YMRS), Severity of Dependence Scale (SDS), and Work and Social Adjustment Scale (WSAS), which are clinically approved tools selected to cover the main disorders in the personality, attitude, behavior, and cognitive status.

### 2.2. Study Population

**Figure 1:**
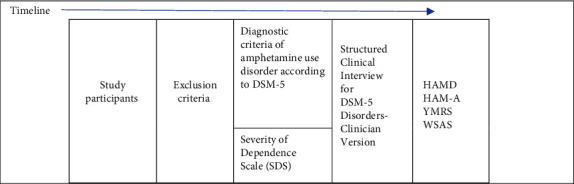
Design of the study (timeline). The initial sample consisted of 110 patients. After applying the exclusion criteria, 35 eligible patients were recruited to participate in this study. Hamilton Depression Rating Scale (HAM-D), Young Mania Rating Scale (YMRS), Severity of Dependence Scale (SDS), and Work and Social Adjustment Scale (WSAS) were administered.

The study population included amphetamine addicts or its derivatives such as methamphetamines, crystals, or others. They were hospitalized in rehabilitation and treatment centers of addiction in public healthcare facilities in Saudi Arabia and were willing to participate in the assessment study. Patients were interviewed by an experienced psychiatrist and were evaluated according to the DSM-5 criteria (Figure 1). Interviews were administered face-to-face. The addicts who were unwilling to participate due to any reason were excluded from this study. Moreover, the study included twenty-five participants who were healthy volunteers. The data from healthy participants were considered as control. The control group participants were frequency-matched to the exposed group in terms of lifestyle and age. All participants were free of any underlying medical condition and had similar dietary habits. The duration of participant recruitment, assessment, and follow-up is one week each.

### 2.3. Inclusion Criteria

The inclusion criteria are as follows:

- Patients with positive amphetamine abuse by detection test and the review of history
- Patients who are ≥18 years and ≤60 years old

### 2.4. Exclusion Criteria

The exclusion criteria are as follows:

- Patients who are not addicted to investigated drugs
- Patients who have disorders that might affect the assessment
- History of psychiatric disorders such as depression, anxiety, mania, schizophrenia, and bipolar disorders prior to addiction
- Patients who are less than 18 years and more than 60 years old

### 2.5. Questionnaire Design and Distribution

Psychiatric assessment tools were used, and a designed bilingual (English and Arabic) electronic questionnaire was developed and utilized to collect the required data from addicts. Complete medical examination and review of patient files (by retrieving the data through computerized health information records) were performed. Data were collected directly from all addicts while they were hospitalized. The assessment tools that have been used to assess and grade the severity of psychiatric disorders such as anxiety, depression, and mania include the following: Hamilton Depression Rating Scale (HAM-D), Hamilton Anxiety Rating Scale (HAM-A), Young Mania Rating Scale (YMRS), Severity of Dependence Scale (SDS), and Work and Social Adjustment Scale (WSAS). A group of psychiatric specialists and toxicology experts reviewed and provided feedback on the questionnaire's initial draft and the beneficial suggestions were incorporated into the approved questionnaire. During this study, all the participants were asked about the clarity, understandability, and relevance of all the questions and response options of the study tool. The final version of the validated questionnaire consisted of 2 sections.  Section 1 comprised of questions related to demographics.  Section 2 had questions assessing the severity of psychiatric disorders by using multiassessment tools approved and used in the psychology and toxicology field.

### 2.6. Assessment Tools during Examination

#### 2.6.1. Severity of Dependence Scale (SDS)

The SDS is a trustworthy tool with strong diagnostic performance that can determine the severity of drug abuse and dependency. It is used to provide a quick assessment of the psychological effects of amphetamine abuse. The SDS is a 5-point questionnaire that gives an overview of the severity of the substance use disorder dependency. The overall score is achieved by adding a 5-point score. That is, the more the reliance, the higher the ranking. The SDS takes less than a minute to complete. In our study, SDS was assessed for each participant on admission before completing the detoxification phase through these questions to the participants: Did you believe that your usage of amphetamine was not under control? Did it make you nervous or frightened to think about missing a fix (or dose) or not pursuing? Did your use of amphetamine cause you any concern? Could you have wished to stop? How challenging would it be for you to quit using amphetamine or go without it? [14].

#### 2.6.2. The Hamilton Depression Rating Scale (HAM-D)

It will be used to grade the depression in the cases selected by accessing mood, guilt feelings, thoughts of suicide, sleeplessness, agitation or retardation, anxiety, weight loss, and physical symptoms. The Hamilton Depression Rating Scale (HAM-D), it will be used to grade the depression in the cases selected by accessing mood, guilt feelings, thoughts of suicide, sleeplessness, agitation or retardation, anxiety, weight loss, and physical symptoms. HAM-D is the most widely used clinician-administered depression assessment scale that contains 17 items pertaining to the symptoms of depression experienced over the past week. It is used to provide an indication of depression and also as a guide to evaluate recovery. The scoring on HAM-D is as follows: 0–7 (no depression), 8–17 (mild depression), 18–23 (moderate depression), and 24 and above (severe depression) [15].

#### 2.6.3. The Hamilton Anxiety Rating Scale (HAM-A)

The anxiety will be evaluated and ranked by looking for two types of anxiety: “somatic anxiety,” which includes general somatic symptoms (sensory) such as cardiovascular, respiratory, gastrointestinal, genitourinary, and autonomic and “psychic anxiety,” which includes anxious mood, tension, fears, insomnia, intellectual (cognitive changes), depressed mood, and behavior during the interview. It is a clinician-rated evaluation composed of 14 items. The scores on this scale range from 0–56 illustrated as follows: 14–17 (mild anxiety), 18–24 (moderate anxiety), and 25–30 (severe anxiety) [16].

#### 2.6.4. Young Mania Rating Scale (YMRS)

In order to determine the degree of manic symptoms, the YMRS measures the following: motor activity, mood, disruptive/aggressive behavior, speech, thinking content, irritability, aggression, critical capacity, aggressiveness, libido, sleep, and overall attitude. YMRS is a clinician-rated scale consisting of 11 item. Four of the YMRS items are rated on a zero to eight scale, with the remaining five items being rated on a zero to four scale. A score of 12 or more was taken as suggestive of the presence of mania [17].

#### 2.6.5. The Work and Social Adjustment Scale (WSAS)

It is a five-item scale measuring the impact of mental health problems on functioning at work, at home, during social and private leisure activities, and in family relationships. This outcome measure is both sensitive and practical, exhibiting connections with the intensity of depression and certain symptoms of anxiety [18].

### 2.7. Urine Collection and Storage

Each urine void will be collected in a polypropylene container and refrigerated immediately after urination at −20°C until analysis.

#### 2.7.1. Determination of Urinary Amphetamine Level

Urine specimens were obtained promptly at the emergency department and then tested for amphetamine abuse by inserting the multidrug screen kit into the urine and waiting 5 minutes to see if it was positive or negative for amphetamine. If the result is positive, we send the urine sample to a laboratory for quantitative evaluation of amphetamine levels in the urine. The DOAULT biochips were supplied as a kit (EV 4101, Randox Laboratories, Crumlin, United Kingdom) containing a calibration CD, 4 cassettes of 10 carriers (with 9-biochip carrier), a diluent, and a conjugate. The drug concentrations for each calibration level and target analytes is described by calibration curves. The biochip was analyzed using the Evidence analyzer (Randox Laboratories, Crumlin, United Kingdom), which takes a 9-biochip carrier through different stages of the assay. The components of the analyzer are described in the previous study [19].

### 2.8. Statistical Analysis

Data were analyzed using the Statistical Package for Social Studies (SPSS) software version 21.0 (SPSS Inc., Chicago) package. A descriptive analysis was carried out using frequencies and percentages for categorical data or means with standard deviation for continuous data, which were first tested for normality by the Shapiro–Wilk test and they were normally distributed. Then, inferential statistics were applied. Independent *T*-test was applied for comparing between the two independent amphetamine and control groups regarding the ages of the participants, as well as the HAM-A, HAM-D, and YMRS, which showed normal distribution between both groups. Pearson correlations were employed to investigate the linear relationship between the studied psychiatric scales and Severity of Dependence Scale, amphetamine levels, age of amphetamine addicts, and the duration of amphetamine intake. Multiple regression analyses were performed to develop models for predicting the Work and Social Adjustment Scale and grades of depression (HAM-D), anxiety (HAM-A), and mania (YMRS). We entered SDS, amphetamine levels, and duration of intake as basic candidate variables for developing the models, and the studied psychiatric scales were tested for multicollinearity by Pearson correlations to decide which scales would be entered as candidate predictors. Multicollinearity was detected between HAM-A and HAM-D (*r* coefficient = 0.888). Furthermore, the stepwise method was applied to include the predictors which contribute significantly to the model. Results were reported as beta coefficients and 95% confidence intervals in addition to the adjusted *R* square. The level of statistical significance was considered at *P* < 0.05. Post hoc power analysis was calculated by G power 3.1.9.4 software program according to the following assumptions: a large effect size of 0.5, defined as the difference between the amphetamine and control groups regarding the studied psychiatric scales, alpha error of 0.05, two-tailed analysis, and the recruited total sample size of 60. The power of the study was 99%.

### 2.9. Ethical Statement

The study was approved by the Institutional Review Board at the Ministry of Health, Qassim Health Cluster, KSA (IRB reference no. 607/44/12773-Mar 20-2023). Informed consent was obtained from the participants before starting the assessment. Confidentiality of data was assured for all the participants using anonymous identifiers.

## 3. Results

### 3.1. Sociodemographic Characteristics

#### 3.1.1. Age

In the present study, the ages of the participants ranged from 18 to 60 years old with mean ages of 38.68 ± 8.14 and 37.77 ± 10.95 years in the control and amphetamine groups, respectively.  Table 1 shows that the majority of participants' ages ranged between 26 and 45 years old (represented 56% and 74.3% in the control and amphetamine groups, respectively), while the lowest percentage of participants had their ages ranged from 18 to 25 years old (represented 4% and 5.7%, in the control and amphetamine groups, respectively). There were no significant changes between the control and amphetamine groups with regard to age range (*P* > 0.05).

#### 3.1.2. Marital Status

Regarding the distribution of the studied participants according to their marital status, the present study showed that the majority of participants were married (80% and 54.3% in the control and amphetamine groups, respectively), while divorced participants were the lowest percentage representing 4% and 8.6% in the control and amphetamine groups, respectively, as shown in Table 2.

#### 3.1.3. Social Status

In the present study, most of the studied participants were living with their families (88% and 85.7% in the control and amphetamine groups, respectively). The percentages of participants living alone in the control and amphetamine groups were 4% and 8%, respectively. However, the percentages of participants living with friends in the control and amphetamine groups were 8.6% and 5.7%, respectively (Table 2).

#### 3.1.4. Occupational Status

As demonstrated in Table 2, the highest percentage of the control group had jobs (96%), while in the amphetamine group, the majority were jobless (Table 2).

#### 3.1.5. Special Habits

The present study showed that all participants were current cigarette smokers and caffeine users (Table 2).

#### 3.1.6. Duration of Intake

The duration of intake in the amphetamine group ranged from 1 to 13 years with a mean duration of 8.34 ± 3.79 years. The majority of patients were addicts for a period of more than 5 years (71.4%) (Table 3).

#### 3.1.7. Motives for Substance Intake

Regarding the motives for intake of the studied patients, the influence of friends represented the most common cause for starting and continuing (54.3%). In addition, lifestyle stress, frustration, and relationship difficulties were important reasons for substance use disorder (11.4%, 14.3%, and 11.4%, respectively). Other reasons for substance use disorders such as enhanced performance and experimental represented the lowest percentages of 5.8% and 2.9%, respectively (Table 4).

#### 3.1.8. Amphetamine Levels and Severity of Dependence Scale (SDS)

The present study demonstrated that the amphetamine levels were 1672.2 ± 295.65 (ng/mL). The mean severity dependence scale value was 10.46 ± 1.82 (Table 5).

### 3.2. Psychiatric Evaluation Parameters

#### 3.2.1. Comparison between the Studied Amphetamine and Control Groups regarding the Studied Scales

**Figure 2:**
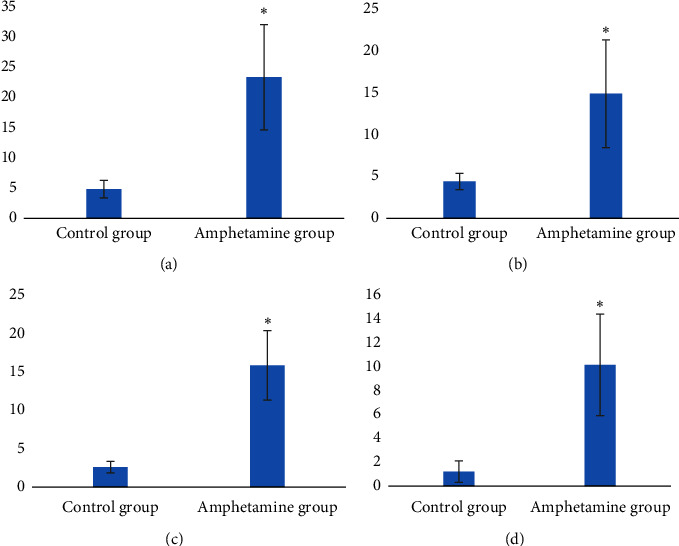
(a) Levels of anxiety according to the Hamilton Anxiety Rating Scale (HAM-A), (b) levels of depression according to the Hamilton Depression Rating Scale (HAM-D), (c) levels of mania according to the Young Mania Rating Scale (YMRS), and (d) Work and Social Adjustment Scale (WSAS) scoring among control and amphetamine groups (*n* = 60). ^*∗*^Significant at *P* < 0.05 level.

In the present study, the mean value of HAM-A scale was significantly higher in the amphetamine group compared to the control group (Figure 2(a)). Likewise, the mean HAM-D value in the amphetamine group was significantly higher than in the control group (Figure 2(b)). The mean MRS also showed a significantly higher mean in the amphetamine group compared to the control group (Figure 2(c)). Regarding the WSAS, Figure 2(d) shows a significantly higher mean in the amphetamine group than in the control group. A significantly higher mean in the amphetamine group was observed.

The mean values of the HAM-A scale were 12.60 ± 0.55, 16.00 ± 1.15, 21.82 ± 2.23, and 33.33 ± 5.45 for participants in the amphetamine group suffering from nonanxiety, mild anxiety, moderate anxiety, and severe anxiety, respectively. On the other hand, the mean values of the HAM-D scale were 6.33 ± 0.58, 11.95 ± 2.65, 19.63 ± 1.51, and 26.00 ± 1.00 for participants in the amphetamine group suffering from nondepression, mild depression, moderate depression, and severe depression, respectively.

### 3.3. Correlations of the Studied Parameters

#### 3.3.1. Hamilton Anxiety Rating Scale (HAM-A)

Table 6 reveals that HAM-A values had a significant correlation with SDS and amphetamine levels in the amphetamine group. On the other hand, it showed no significant correlations with age and duration of addiction in the amphetamine group.

#### 3.3.2. Hamilton Depression Rating Scale (HAM-D)

Table 6 reveals that HAM-D values had a significant correlation with SDS and amphetamine levels in the amphetamine group, while HAM-D values showed no significant correlations with age and duration of addiction in the amphetamine group.

#### 3.3.3. Young Mania Rating Scale (YMRS)

Table 6 reveals that YMRS values had a significant correlation with SDS, amphetamine levels, and duration of addiction in the amphetamine group. YMRS had no significant correlations with age in the amphetamine group.

#### 3.3.4. The Work and Social Adjustment Scale (WSAS)

Table 6 reveals that WSAS values had significant correlations with SDS and amphetamine levels in the amphetamine group. WSAS had no significant correlations with age and duration of addiction in the amphetamine group.

### 3.4. Multiple Regression Models

#### 3.4.1. Prediction of the Work and Social Adjustment Scale

The model was a good fit for the data and significantly predicted the outcome (*P* < 0.001). The anxiety scale was the only variable that contributed significantly to predicting the Work and Social Adjustment Scale. For every unit increase in the HAM-A, the WSAS scales increase by 0.414 (CI: 0.338–0.490). The model explained 77.0% of the variability in the Work and Social Adjustment Scale (adjusted *R*^2^) (Table 7).

#### 3.4.2. Prediction of the Depression Grade (HAM-D)

The model was a good fit for the data and significantly predicted the outcome (*P* < 0.001). The amphetamine level was the only variable that contributed significantly to predicting the HAM-D. For every unit increase in the amphetamine level, the HAM-D increases by 0.014(CI: 0.008–0.020). The model explained 57.5% of the variability in the HAM-D (adjusted *R*^2^) (Table 7).

#### 3.4.3. Prediction of the Anxiety Grade (HAM-A)

The model was a good fit for the data and significantly predicted the outcome (*P* < 0.001). The amphetamine level was the only variable that contributed significantly for predicting the HAM-A. For every unit increase in the amphetamine level, the HAM-A increases by 0.018(CI: 0.012–0.023). The model explained 53.5% of the variability in the HAM-A (adjusted *R*^2^) (Table 7).

#### 3.4.4. Prediction of the Mania Grade (YMRS)

The model was a good fit for the data and significantly predicted the outcome (*P* < 0.001). Severity of addiction (SDS) was the only variable that contributed significantly for predicting the YMRS. For every unit increase in SDS, the YMRS increases by 1.385 (CI: 0.850–1.921). The model explained 42.5% of the variability in the YMRS (adjusted *R*^2^) (Table 7).

## 4. Discussion

Psychiatric disorders are substantially accompanied by drug abuse, especially after a long period of abuse of specific illicit drugs. The severity of psychiatric disorders among addicts depends on multiple factors such as type of abused drugs, abuse duration, materials adulteration, abuse methods, amount, genetic susceptibility, and coingestion of multiple drugs. Early detoxification, coping, and prevention can minimize the complication and progression of psychosis and help the addicts to join society and become influential members. Time is a cornerstone in the process of psychiatric illness development [20–22].

The underlying mechanisms of psychosis among addicts are multiple and subjected to direct and indirect factors. Most studies have shown that drug abuse affects the functions and structures of the brain and results in damage and ultimately, psychiatric disorders. Most of the investigated mechanisms revolve around monoamines such as dopamine, epinephrine, norepinephrine, and serotonin and their level within the neuronal synapse. Dopamine and norepinephrine released inside the nucleus accumbens are linked to euphoria and reward feedback loop, which cause an addiction. Direct mechanisms can induce psychosis due to the toxicity of the dopamine system, glutamate, and GABA by abused drugs. Indirect mechanisms involved include drug type, long-term abuse, dosage, sleep disorders, additives adulterations, and reactive oxygen species. Other mechanisms investigated to induce psychosis among drug abusers include neuronal inflammation, neurotoxicity, apoptosis, brain network dysregulation, and mediators of protein kinases [23–27].

This is the first study of its kind to compare the demographics, psychiatric disorders, and functioning and performance for work and social demands between amphetamine addicts in Saudi Arabia by using multiassessment tools that are approved in the psychology field. The results of the present study demonstrated that subjects aged between 26 and 45 years are the most prevalent class to abuse amphetamines. These results are consistent with the result of a study carried out in 2018 in the Qassim region, KSA, in which amphetamine abuse was most predominant in 20–40 year-olds [28].

Living with family may not be a barrier to preventing addiction in our study because we found that 85.7% of addicts were living with their families. These findings explain the default in the family role in controlling, monitoring, caring, advising, and guidance of addicts. Islamic guidance is important for the prevention and control of drug abuse and should be the cornerstone to educate the offspring about the advice and recommendations since childhood [29]. Each culture has distinct beliefs and unique ways of raising children. Cultural differences in parenting beliefs and behaviors are an interesting area that enhances understanding of the nature of differences across cultures. Substance abuse risk may be related to family sociocultural factors. In a study of sociocultural factors, sensation seeking, and risk of exposure to substance abuse among Egyptian and Saudi undergraduates, the risk for substance abuse in both cultural settings was moderate, and smoking was the most common substance used. Moreover, a highly supportive paternal relationship reduced the risk of drug involvement by 85% [30].

Most participants in the amphetamine group of our study are jobless due to reasons that may be related to addiction (77.1%). Unemployment is a direct reason for pushing the young generation to addiction, and this gives an indicator that employing youth in suitable work and beneficial activities will help to minimize the addiction phenomenon. The addicts of amphetamines are vulnerable to unemployment or termination, which may be the result of the aggressive behavior of abusers of this class of drugs. This is in accordance with another study which demonstrated that unemployment was associated with substance abuse admissions in the rehabilitation center for drugs of abuse, and they claim that economic hardship is linked to increased substance abuse [28, 29, 31].

According to a report of healthcare workers in the rehabilitation centers in the USA, there are increases in the prevalence of rare psychiatric symptoms in young people, which is considered a strange issue and for unknown reasons. An adulteration and malpractice in the manufacturing processes of these substances may be implicated in the appearance of psychosis in the young people. According to a study that tested illicit drugs for adulterants, they found that in some abused substances, more than 80% contained additional substances, including fentanyl, cocaine, heroin, and more [32].

The duration of intake in the studied addicts ranged from 1 to 13 years with a mean duration of around 7 years. Amphetamines are highly addictive drugs and the study reflects persistent susceptibility to addiction and psychosis in the long term on this harmful class of abused drugs. Around 64% of amphetamine addicts have been consuming these stimulants for more than 10 years and the high patient rate seeking detoxification treatment in rehabilitation centers reflects the amount of damage to the mental status of addicts. According to a study investigating the effect of long-period addiction, most patients with substance-induced psychotic disorders had a good long-term prognosis, but those who started illegal drug use early, used drugs for prolonged periods or had a family history of psychiatric illnesses and were more likely to develop a chronic psychosis, which may reflect the neurotoxic effects of these abused drugs [33].

The present study investigated the motivations for abuse and found that friends influence represented the most common motive of amphetamine abuse. The other important findings such as life stressors, free time, frustration, social affairs, enhancement, experimental desire, and relationship difficulties were additional motivations for substance abuse. The present study also showed that cigarette smoking is highly prevalent among the addicts. Another study investigated the motivations and risks of abusing illicit drugs and they found that there are individual risk factors identified such as high impulsivity, rebelliousness, emotional regulation impairment, low religiosity, pain catastrophic, major depressive disorder, behavioral addiction, low-perceived risk, and high-attitude to use synthetic drugs [29, 34].

The present study demonstrated that mood disturbances, behavior-cognitive abnormalities, and bipolar risk are correlated with high amphetamine dependency, high doses, and a long period of addiction. These findings are most probably a result of brain damage, neurotoxicity, neuronal inflammation, dopamine sensitization, and network dysregulation, which are linked to amphetamine abuse.

Despite that mood disorders increase the risk of substance abuse, the converse is also possible. Chronic substance abuse sometimes “unmasks” bipolar or other mood disorders. It triggers an increase in symptom severity from a subclinical to a clinically significant level. This appears to occur because in genetically vulnerable individuals, the drugs exacerbate pathophysiological changes in neurotransmitter systems or signaling pathways that already are abnormal and underlie the mood disorder. Another proposed explanation for the high comorbidity rate of mood disorders with SUDs involves “kindling.” The term, usually associated with epilepsy, refers to the concept that repeated disruptions, such as those that occur during seizures, sensitize brain cells. The more sensitized the neurons become, the less it takes to disrupt them, which is why in untreated epilepsy, seizures tend to become more frequent and severe over time. Both alcohol and cocaine sensitize neurons, and this increased sensitivity may contribute to the typical progression from occasional to increasingly frequent and intense use of these substances. Mood disorders often follow a similar course of increasingly distressing symptomatic episodes separated by progressively shorter periods of remission, suggesting that they too may intensify via a kindling process. The kindling explanation for comorbidity, then, holds that in vulnerable individuals, an underlying neurobiological tendency to sensitization may promote both drug dependence and mood disorders. [35].

Our results are consistent and supported by the results of Quello et al., 2005, who demonstrated that chronic use of central nervous system (CNS) stimulants, such as cocaine and amphetamines, may produce symptoms that are typical of bipolar spectrum disorders, such as euphoria, increased energy, decreased appetite, grandiosity, and paranoia [20]. Psychotic symptoms such as delusions and hallucinations are reported to be increased among stimulant abuse such as amphetamines and MDMA. According to studies, they concluded that patients with drug-induced psychosis were more likely to develop a schizophrenia-spectrum disorder [36].

The results of the present study also demonstrated that depression and anxiety did not correlate significantly with the period of addiction, which explains the endurance of coping with these feelings by addicts. These results are consistent with the results of another study carried out in Malaysia, which assessed anxiety and depression among the amphetamines group. It involved 215 abusers and found anxiety in around 44% of them (*n* = 96) and depression in around 50% of them (*n* = 108), and the investigators claimed that subjects with higher religiosity and positive religious coping were less anxious or depressed, and they are found that negative religious coping was significantly associated with anxiety and depression in amphetamines abusers [21].

## 5. Conclusions

This study emphasized that amphetamine addiction significantly hampers a person's ability to meet the demands of work and social functioning while also having a detrimental effect on their mental health. The risk of developing anxiety, depression, and mood disturbances due to amphetamine abuse is higher in comparison to individuals who are nonaddicts. These adverse effects can be attributed to brain damage, neurotoxicity, neuronal inflammation, and dysregulation of brain networks. These abnormalities are particularly evident with prolonged abuse and higher doses of amphetamine and its derivatives.

## Figures and Tables

**Table 1:** Distribution of the control and amphetamine groups (*n* = 60) according to their ages (years).

Age groups (years)	Control group (*n* = 25)			Amphetamine group (*n* = 65)		
	No.	%	Mean ± SD	No.	%	Mean ± SD
18–25	1	4	38.68 ± 8.14	2	5.7	37.77 ± 10.97
26–35	10	40		18	51.4	
36–45	4	16		8	22.9	
> 45	3	12		7	20	

**Table 2:** Distribution of the control and amphetamine groups (*n* = 60) according to their marital status, living status, social status, and occupational status.

Items		Control group (*n* = 25)	Amphetamine group II (*n* = 35)
Marital status (%)	Single	16	37.1
	Married	80	54.3
	Divorced	4	8.6

Living status (%)	Living alone	4	8.6
	Living with family	88	85.7
	Living with friends	8	5.7

Occupational status (%)	Student	0	5.8
	Employee	96	17.1
	Unemployed	4	77.2

Special habits (%)	Smoker	100	100
	Caffeine user	100	100

**Table 3:** Distribution of the amphetamine group (*n* = 35) according to the duration of intake (in years).

Duration of intake (years)	Amphetamine group (*n* = 35)		
	No.	%	Mean ± SD
2-3 years	3	8.6	8.34 ± 3.79
4-5 years	7	20	
More than 5 years	25	71.4	

**Table 4:** Distribution of the amphetamine group (*n* = 35) according to motives for intake.

Motives for substance intake	Amphetamine group (*n* = 35)	
	No.	%
Lifestyle stress	4	11.4
Influence of friends	19	54.3
Frustration	5	14.3
Relationship difficulties	4	11.4
Enhance performance	2	5.8
Experimental	1	2.9

**Table 5:** Amphetamine level and Severity of Dependence Scale (SDS) for the amphetamine group (*n* = 35) prior to treatment.

Items	Amphetamine group (n = 35)
Amphetamine level (ng/ml)	1672.2 ± 295.65
CIWA-Ar	10.46 ± 1.82

**Table 6:** Pearson correlation coefficients between Severity of Dependence Scale (SDS), amphetamine level, age, duration of addiction, and Hamilton Anxiety Rating Scale (HAM-A), Hamilton Depression Rating Scale (HAM-D), Young Mania Rating Scale (YMRS), and the Work and Social Adjustment Scale (WSAS) among the amphetamine group.

Items	SDS	Amphetamine level	Age	Duration of addiction
HAM-A	574^*∗∗*^	759^*∗∗*^	−0.185	0.075
HAM-D	597^*∗∗*^	825^*∗∗*^	−0.024	0.184
YMRS	588^*∗∗*^	569^*∗∗*^	0.018	0.366^*∗*^
WSAS	430^*∗∗*^	693^*∗∗*^	−0.071	0.046

^*∗*^Correlation was statistically significant at 0.05 level (2-tailed). ^*∗∗*^Correlation was statistically significant at 0.01 level (2-tailed).

**Table 7:** Stepwise multiple regression analyses for developing models to predict Work and Social Adjustment Scale and grades of depression (HAM-D), anxiety (HAM-A), and mania (YMRS).

Items	Unstandardized *B* coefficients	95% CI of *B*	*P* value of the predictor	Adjusted *R*^2^ (%)	ANOVA *P* value of the model
*Prediction of the Work and Social Adjustment Scale*					
HAM-A	0.414	0.338–0.490	<0.001^*∗*^	77.0	<0.001^*∗*^
Constant	0.246		0.790		

*Prediction of the depression grade (HAM-D)*					
Amphetamine levels	0.014	0.008–0.020	<0.001^*∗*^	57.5	<0.001^*∗*^
Constant	−4.608		0.110		

*Prediction of the anxiety grade (HAM-A)*					
Amphetamine levels	0.018	0.012–0.023	<0.001^*∗*^	53.5	<0.001^*∗*^
Constant	−6.250		0.178		

*Prediction of the mania grade (YMRS)*					
SDS	1.385	0.850–1.921	<0.001^*∗*^	42.5	<0.001^*∗*^
Constant	1.143		0.681		

^*∗*^Significant at *P* < 0.05. CI, confidence interval.

## Data Availability

The data used to support the findings of this study are available from the corresponding author upon request.

## References

[1] McKetin R., Lubman D. I., Baker A. L., Dawe S., Ali R. L. (2013). Dose-related psychotic symptoms in chronic methamphetamine users: evidence from a prospective longitudinal study. *JAMA Psychiatry*.

[2] Alharbi R. S., Alhowail A. H., Alharbi A. G., Emara A. M. (2022). Evaluation of the health status outcome among inpatients treated for amphetamine addiction. *Saudi Journal of Biological Sciences*.

[3] Robinson T. E., Berridge K. C. (2000). The psychology and neurobiology of addiction: an incentive-sensitization view. *Addiction*.

[4] Bramness J. G., Gundersen Ø. H., Guterstam J. (2012). Amphetamine-induced psychosis--a separate diagnostic entity or primary psychosis triggered in the vulnerable?. *BMC Psychiatry*.

[5] Mullen J. M., Richards J. R., Crawford A. T. (2023). *Amphetamine-Related Psychiatric Disorders*.

[6] McKetin R., Leung J., Stockings E. (2019). Mental health outcomes associated with the use of amphetamines: a systematic review and meta-analysis. *EClinicalMedicine*.

[7] Leung J., Mekonen T., Wang X., Arunogiri S., Degenhardt L., McKetin R. (2023). Methamphetamine exposure and depression-A systematic review and meta-analysis. *Drug and Alcohol Review*.

[8] Hilty D. M., Leamon M. H., Lim R. F., Kelly R. H., Hales R. E. (2006). A review of bipolar disorder in adults. *Psychiatry*.

[9] Snyder S. H. (1973). Amphetamine psychosis: a model schizophrenia mediated by catecholamines. *American Journal of Psychiatry*.

[10] Bell D. S. (1973). The experimental reproduction of amphetamine psychosis. *Archives of General Psychiatry*.

[11] Wodarz N., Krampe-Scheidler A., Christ M. (2017). Evidence-based guidelines for the pharmacological management of acute methamphetamine-related disorders and toxicity. *Pharmacopsychiatry*.

[12] Ujike H., Sato M. (2004). Clinical features of sensitization to methamphetamine observed in patients with methamphetamine dependence and psychosis. *Annals of the New York Academy of Sciences*.

[13] Grelotti D. J., Kanayama G., G Pope H. (2010). Remission of persistent methamphetamine-induced psychosis after electroconvulsive therapy: presentation of a case and review of the literature. *American Journal of Psychiatry*.

[14] Gossop M., Darke S., Griffiths P. (1995). The Severity of Dependence Scale (SDS): psychometric properties of the SDS in English and Australian samples of heroin, cocaine and amphetamine users. *Addiction*.

[15] Hamilton M. (1960). A rating scale for depression. *Journal of Neurology, Neurosurgery and Psychiatry*.

[16] Hamilton M. (1959). The assessment of anxiety states by rating. *British Journal of Medical Psychology*.

[17] Young R. C., Biggs J. T., Ziegler V. E., Meyer D. A. (1978). A rating scale for mania: reliability, validity and sensitivity. *British Journal of Psychiatry*.

[18] Mundt J. C., Marks I. M., Shear M. K., Greist J. M. (2002). The work and social adjustment scale: a simple measure of impairment in functioning. *British Journal of Psychiatry*.

[19] Schumann G., Klauke R. (2003). New IFCC reference procedures for the determination of catalytic activity concentrations of five enzymes in serum: preliminary upper reference limits obtained in hospitalized subjects. *Clinica Chimica Acta*.

[20] Quello S. B., Brady K. T., Sonne S. C. (2005). Mood disorders and substance use disorder: a complex comorbidity. *Science and Practice Perspectives*.

[21] Chok How T., Abd Rashid R., Chong Guan N. (2020). Anxiety and depression among amphetamine-type stimulant users: the association with religiosity and religious coping. *Malaysian Journal of Medical Sciences*.

[22] Treatment for Stimulant Use Disorders (1999). Rockville (MD): substance abuse and mental health services administration (US).

[23] Koob G. F., Volkow N. D. (2016). Neurobiology of addiction: a neurocircuitry analysis. *The Lancet Psychiatry*.

[24] Volkow N. D., Michaelides M., Baler R. (2019). The neuroscience of drug reward and addiction. *Physiological Reviews*.

[25] Bromberg-Martin E. S., Matsumoto M., Hikosaka O. (2010). Dopamine in motivational control: rewarding, aversive, and alerting. *Neuron*.

[26] Müller C. P., Homberg J. R. (2015). The role of serotonin in drug use and addiction. *Behavioural Brain Research*.

[27] Volkow N. D., Morales M. (2015). The brain on drugs: from reward to addiction. *Cell*.

[28] Ibrahim Y., Hussain S. M., Alnasser S., Almohandes H., Sarhandi I. (2018). Patterns and sociodemographic characteristics of substance abuse in Al Qassim, Saudi Arabia: a retrospective study at a psychiatric rehabilitation center. *Annals of Saudi medicine*.

[29] Nawi A. M., Ismail R., Ibrahim F. (2021). Risk and protective factors of drug abuse among adolescents: a systematic review. *BMC Public Health*.

[30] Neama F. (2021). Sociocultural factors, sensation seeking, and risk of exposure to substance abuse among Egyptian and Saudi undergraduates the open. *Psychology Journal*.

[31] Azagba S., Shan L., Qeadan F., Wolfson M. (2021). Unemployment rate, opioids misuse and other substance abuse: quasi-experimental evidence from treatment admissions data. *BMC Psychiatry*.

[32] Singh V. M., Browne T., Montgomery J. (2020). The emerging role of toxic adulterants in street drugs in the US illicit opioid crisis. *Public Health Reports*.

[33] Deng X., Huang Z., Li X. (2012). Long-term follow-up of patients treated for psychotic symptoms that persist after stopping illicit drug use. *Shanghai Archives of Psychiatry*.

[34] Whitesell M., Bachand A., Peel J., Brown M. (2013). Familial, social, and individual factors contributing to risk for adolescent substance use. *Journal of Addiction*.

[35] Pettinati H. M., O’Brien C. P., Dundon W. D. (2013). Current status of co-occurring mood and substance use disorders: a new therapeutic target. *American Journal of Psychiatry*.

[36] Fiorentini A., Cantù F., Crisanti C., Cereda G., Oldani L., Brambilla P. (2021). Substance-induced psychoses: an updated literature review. *Frontiers in Psychiatry*.

